# Effect of Salt Substitution on Cardiac Structure and ECG Parameters in Middle-aged and Elderly Hypertensive Patients

**DOI:** 10.31083/j.rcm2511390

**Published:** 2024-10-31

**Authors:** Li Che, Ying Zhang, Xia Chen, Haiqing Du, Wei Song, Yinong Jiang

**Affiliations:** ^1^Department of Cardiology, Central Hospital of Dalian University of Technology, 116033 Dalian, Liaoning, China; ^2^Department of Cardiology, First Affiliated Hospital of Dalian Medical University, 116011 Dalian, Liaoning, China; ^3^Dalian Xigang People Square Shidao Street Community Health Service Center, 116016 Dalian, Liaoning, China; ^4^Dalian Xigang People’s Square Changchun Road Community Health Service Station, 116011 Dalian, Liaoning, China

**Keywords:** blood pressure, salt substitution, sodium, potassium, cardiac

## Abstract

**Background:**

Salt substitution (SS) has been found to reduce blood pressure (BP). However, the impact of SS on cardiac structure, as assessed through ultrasonic cardiogram (UCG) and electrocardiograms (ECG), remains poorly understood. This study aims to evaluate the effects of SS on cardiac structure and ECG parameters.

**Methods:**

This 12-month prospective, multi-center, randomized, double-blind study involved hypertensive patients aged 50 to 70 years with office systolic BP (SBP) ranging from 140 to 180 mmHg and diastolic BP (DBP) ranging from 90 to 110 mmHg. A total of 352 patients were enrolled and equally randomized to either the normal salt (NS) group or SS group. Office BP measurements (OBPM) were obtained at baseline and at 3, 6, and 12 months, while home BP measurements (HBPM) were recorded at baseline, 3, 6, 9, and 12 months. Fasting blood, UCG, and ECG parameters were obtained at baseline and at the end of the study.

**Results:**

Of the 352 enrolled patients, 322 completed the study. In the SS group, the reductions in systolic OBPM, HBPM, and diastolic HBPM were significantly greater than those in the NS group. Notable cardiac parameter changes included a reduction in QT dispersion (QTd) by –5 ms (–10, 5) in the NS group and –5 ms (–15, 0) in the SS group (*p* = 0.001); the T wave peak-to-end (Tp-e) value was 0 ms (–5, 10) and –5 ms (–10, 0) (*p* < 0.001), respectively; and Tp-e/QT was 0 (–0.01, 0.02) and –0.02 (–0.04, 0) (*p* < 0.001), respectively. Additionally, left atrial diameter (LAD) was 0 mm (–1, 1) and –1 mm (–2, 1) (*p* < 0.001), and the change in left ventricular mass (LVM) was –2 g (–17.75, 11) and –7 g (–18, 6) (*p* = 0.035), respectively.

**Conclusions:**

This study demonstrates that SS not only significantly decreased LAD and LVM, indicating a significant effect on cardiac structure, but also improves UCG parameters, including reductions in QTd, Tp-e, and Tp-e/QT. These findings highlight the potential of SS as a beneficial intervention in managing cardiac risks in hypertensive patients.

**Clinical Trial Registration:**

ChiCTR1800019727. (https://www.chictr.org.cn/showproj.html?proj=31036).

## 1. Introduction

In 2010, approximately 1.4 billion people worldwide had hypertension (elevated blood pressure [BP]), representing 31.1% of the global population [1]. In wealthy countries, the prevalence, management, and control rates of hypertension were 67%, 55.6%, and 28.4%, respectively, while in low-and middle-income countries, these rates were 37.9%, 29%, and 7.7% [1]. Hypertension remains the primary cause of death globally, accounting for over 10 million annually deaths [2].

A 3-year randomized, placebo-controlled study demonstrated that a low-salt, high-potassium salt substitution (SS, 65% NaCl, 25% KCl, 10% MgSO_4_) significantly reduced systolic BP (SBP) by 9.19 mmHg and diastolic BP (DBP), by 3.03 mmHg compared to normal salt (NS) [3]. Another study indicated that potassium-enriched SS protected organs such as the heart and kidneys, in hypertensive rats [4]. In addition, echocardiography has demonstrated prognostic value for heart disease in hypertensive patients [5]. Therefore, it is important to evaluate the effect of SS on ultrasonic cardiogram (UCG) parameters.

Many lines of evidence indicate that hypertensive patients have higher QT dispersion parameters compared to those without hypertension [6]. Prolonged QT interval (QT) and corrected QT interval (QTc), whether they were congenital or acquired, were linked to malignant arrhythmias, primarily torsades de pointes (TdP) ventricular arrhythmias, and sudden death in both healthy individuals and patients with cardiovascular disease [7, 8, 9, 10]. However, there is limited data on the relationship between SS and electrocardiogram (ECG) parameters. Hence, this study aimed to evaluate the effect of SS on cardiac structure and ECG parameters in mild-to-moderate middle-aged and elderly hypertensive patients.

## 2. Methods

### 2.1 Participants and Study Design

This was a 12-month prospective, multi-center, randomized, double-blind study. Patients with hypertension were chosen from two Dalian, Liaoning, North China community service centers (Dalian Xigang People Square Shidao Street Community Health Service Center and Dalian Xigang People’s Square Changchun Road Community Health Service Station). Hypertension was defined as office SBP ≥140 mmHg and/or DBP ≥90 mmHg or taking antihypertensive agents [11]. The inclusion criteria were as follows: (1) primary hypertension, which we defined as SBP <180 mmHg and/or DBP <110 mmHg; (2) being aged from 50 to 70 years; (3) eating at least two meals at home every day; and (4) having a serum potassium concentration <5.0 mmol/L at baseline.

The sample size was estimated according to the formula N = (1 + 1⁄κ) (σ$\frac{z\,1-\frac{\alpha }{2}+z\,1-\beta }{\text{µ}\,A-\text{µ}\,B}$)^2^, where the value of α was 0.05 and that of β was 0.1. The sample size was determined to be 160 for both the SS and NS groups. Considering losses to follow-up and other problems, we expanded the participation count by 10%. Thus, we enrolled 352 patients total, with 176 in each group.

### 2.2 Ethics Statement

The Chinese Clinical Trial Registry number for this study is: ChiCTR1800019727. The project received ethical approval from the Human Ethics Committee of Dalian Medical University (DMU). Each participant signed an informed consent form.

### 2.3 Protocol

The protocol involved an initial interview conducted by skilled investigators on demographic traits (age, sex, height, weight, and usage of antihypertensive agents). The formula for calculating body mass index (BMI) was weight divided by squared height. Employing a computerized randomization program, the participants enrolled in the study were randomized into either the NS or SS group in a 1:1 ratio. The NS contained 100% sodium chloride, and the SS consisted of 43% sodium chloride, 32% potassium chloride, while 25% was comprised of other ingredients.

### 2.4 Electrocardiography

Standard 12-lead ECG recordings at a paper speed of 25 mm/s were obtained from patients in the supine position using a commercially available device (NIHON KOHDEN ECG2350, Shanghai, China); the patients were examined at both baseline and the study endpoint. The QT interval was measured manually across three consecutive beats from the QRS complex onset to the terminus of the T wave’s return to the T–P baseline, each of which was performed by an observer blind to the study groups. The arithmetic mean was then used. In cases of U waves, the end was determined by the nadir between the T and U waves, but when the T waves were superimposed on the U waves, this lead was discarded. The corrected QT interval (QTc) was derived using Bazett’s formula from 1920: (QTc = QT interval/$\sqrt[{2}]{R\,R}$) in 1920 [11]. The QT interval difference between the longest and shortest QTs was used to calculate QT dispersion (QTd). The V5 lead’s T wave peak-to end (Tp-e) interval was calculated by measuring the time interval between the T wave’s peak and end. V4 and V6 were used when the V5 lead was not suitable.

### 2.5 Measurement of Blood Pressure

As mentioned in our previous article, office BP measurements (OBPM) was obtained at baseline and the 3rd, 6th, and 12th months, and home BP measurements (HBPM) was obtained at baseline and the 3rd, 6th, 9th, and 12th months [12].

### 2.6 Echocardiograph

The ECG parameters were measured by a doctor with specialized training while the patient was in the supine position (using Mindray DC-N6PRO, Shenzhen, China) at both the baseline and the endpoint of the study. The left atrial diameter (LAD), interventricular septal thickness at end diastole (IVSTd), left interventricular internal diameter at end diastole (LVIDd), and the thickness of the left interventricular posterior wall thickness at end diastole (LVPWTd) were measured using two-dimensional images. Left ventricular mass (LVM) was calculated via the following formula: LVM = 0.8 [1.04 (IVSTd + LVIDd + LVPWTd)^3^ – (LVIDd)^3^] + 0.6. Finally, the LVM index (LVMI) was calculated as LVM/body surface area.

### 2.7 Statistical Analysis

Statistical analysis was performed using SPSS 25.0 (IBM Corp., Armonk, NY, USA). Means ± SDs and *t*-tests were used to express and compare continuous variables with a normal distribution. Median and interquartile ranges and the Mann–Whitney U test were used to describe and compare non-normally distributed variables. Frequency and Chi-squared tests were used to show and compare categorical variables. A value of *p* < 0.05 was considered statistically significant.

## 3. Results

### 3.1 Characteristics of the Participants

A total of 352 patients were recruited at baseline, with 30 (8.5%) withdrawing during the intervention. After completion of the interviews, the NS group had 160 participants, and the SS group had 162 participants. The average ages of the NS and SS group participants were 62.17 ± 4.69 and 62.96 ± 4.51 years, respectively. The NS group included 72 (45.0%) male participants, while the SS group included 58 (35.8%) male participants. There were no statistically significant differences in age, sex, BMI, antihypertension agent usage, or systolic and diastolic OBPM at baseline (Table 1).

**Table 1.** **Baseline demographic and clinical profile of hypertensive participants by treatment group**.

Characteristics		NS	SS	*p*
		(n = 160)	(n = 162)	
Age (y)		62.17 ± 4.69	62.96 ± 4.51	0.125
Male sex (N, (%))		72 (45%)	58 (35.8%)	0.093
BMI (kg/m^2^)		25.67 ± 3.19	26.16 ± 3.23	0.174
Antihypertension agents				
	CCB (N, (%))	106 (66.3)	109 (67.3)	0.844
	ACEI/ARB (N, (%))	58 (36.3)	70 (43.2)	0.131
	β-blocker (N, (%))	29 (18.1)	24 (14.8)	0.423
	ACEI/ARB+CCB (N, (%))	33 (20.6)	42 (25.9)	0.261
	β-blocker+CCB (N, (%))	19 (11.9)	18 (11.1)	0.830
	β-blocker+ACEI/ARB (N, (%))	9 (5.6)	13 (8)	0.393
	ACEI/ARB+β-blocker + CCB (N, (%))	5 (3.1)	8 (4.9)	0.409
Baseline OBPM (mmHg)				
	SBP	133.2 ± 13.0	135.3 ± 13.7	0.161
	DBP	78.0 ± 11.2	77.6 ± 10.1	0.744

NS, normal salt; SS, salt substitution; BMI, body mass index; CCB, calcium channel blocker; ACEI, angiotensin-converting enzyme inhibitor; ARB, angiotensin II receptor blocker; BP, blood pressure; OBPM, office BP measurements; SBP, systolic BP; DBP, diastolic BP.

### 3.2 Comparison of HBPM and OBPM at Baseline and the Endpoint between the NS and SS Groups

**Fig. 1.**
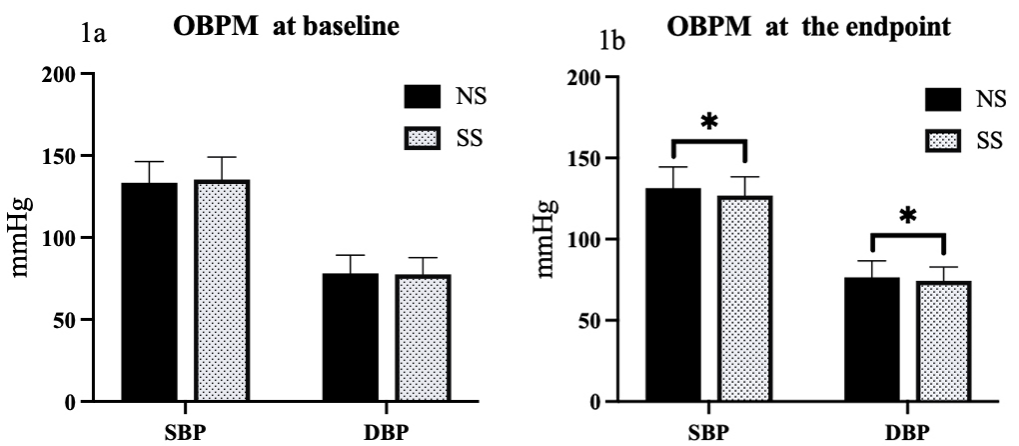
**Comparison of OBPM responses to SS diets in hypertensive patients**. (1a) Comparison of systolic and diastolic OBPMs between NS and SS groups at baseline. (1b) Comparison of systolic and diastolic OBPMs between NS and SS groups at the study endpoint. *p < 0.05. NS, normal salt; SS, salt substitution; BP, blood pressure; OBPM, office BP measurements; SBP, systolic blood pressure; DBP, diastolic blood pressure.

**Fig. 2.**
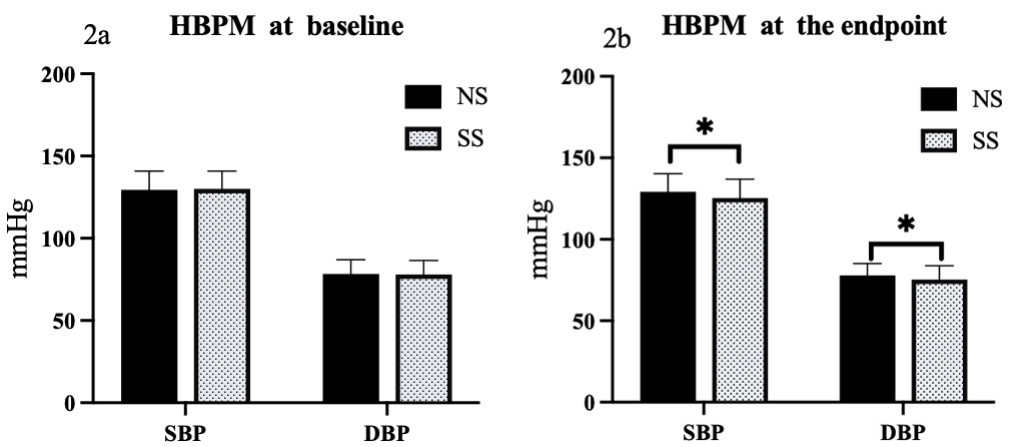
**Comparison of HBPM responses to SS diets in hypertensive patients**. Comparison of home BP measurement (HBPM) between NS and SS groups at baseline (2a) and the endpoint (2b). *p < 0.05. NS, normal salt; SS, salt substitution; BP, blood pressure; SBP, systolic blood pressure; DBP, diastolic blood pressure.

At baseline, there were no significant differences between systolic and diastolic OBPM and HBPM (all *p* > 0.05). However, at the study endpoint, there were significant differences in BP levels. Specifically, both the systolic and diastolic OBPM were significantly lower in the SS group as compared to the NS group (*p* = 0.001 for SBP, and *p* = 0.04 for DBP). There were also significant decreases to both the systolic and diastolic HBPM in the LS group when compared to the NS group (*p* = 0.001 for SBP, and *p* = 0.04 for DBP) (Figs. 1,2).

### 3.3 Ultrasonic Cardiogram Parameters at Baseline and the Endpoint

**Fig. 3.**
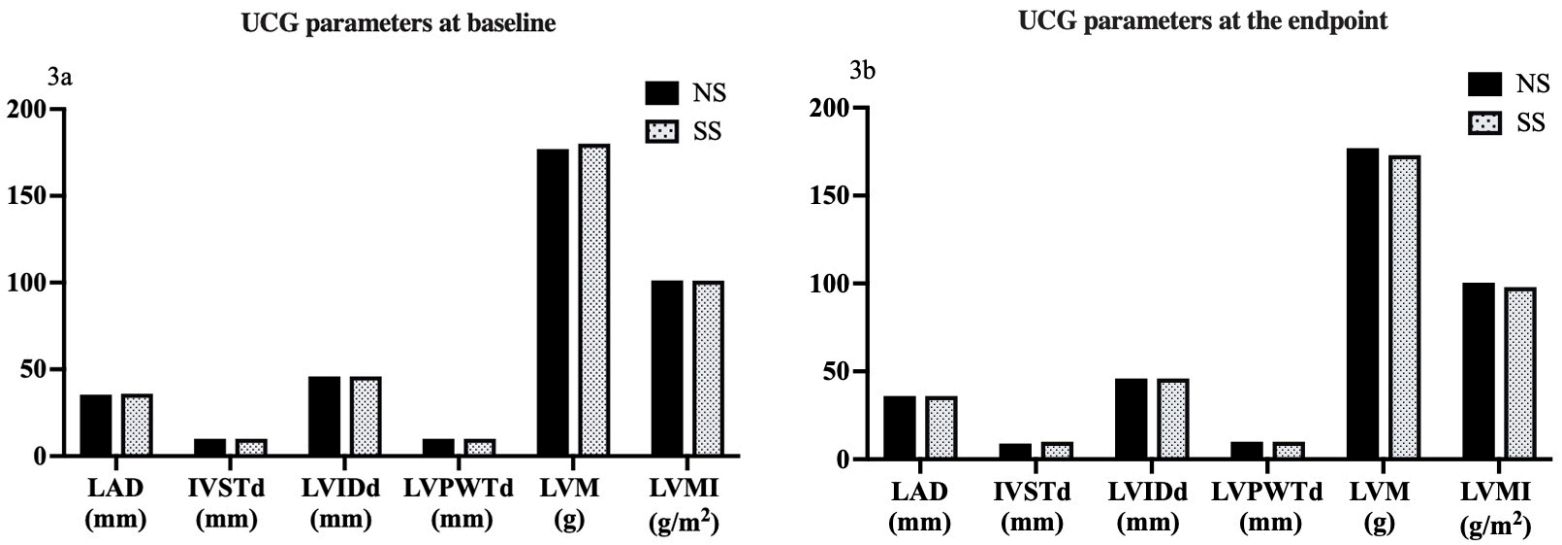
**Comparison of UCG parameters in response to SS diets in hypertensive patients**. (3a) Comparison of UCG parameters at baseline. (3b) Comparison of UCG parameters at the study endpoint. NS, normal salt; SS, salt substitution; UCG, ultrasonic cardiogram; LAD, left atrial dimension; IVSTd, interventricular septal thickness at end diastole; LVIDd, left interventricular internal diameter at end diastole; LVPWTd, left interventricular posterior wall thickness at end diastole; LVM, left ventricular mass; LVMI, left ventricular mass index.

There were no statistical differences in LAD, IVSTd, LVIDd, LVPWTd, LVM, and LVMI between the two groups at baseline and the end of the study (all *p* > 0.05) (Fig. 3).

### 3.4 Comparison of Changes in UCG Parameters between the NS and SS Groups

**Fig. 4.**
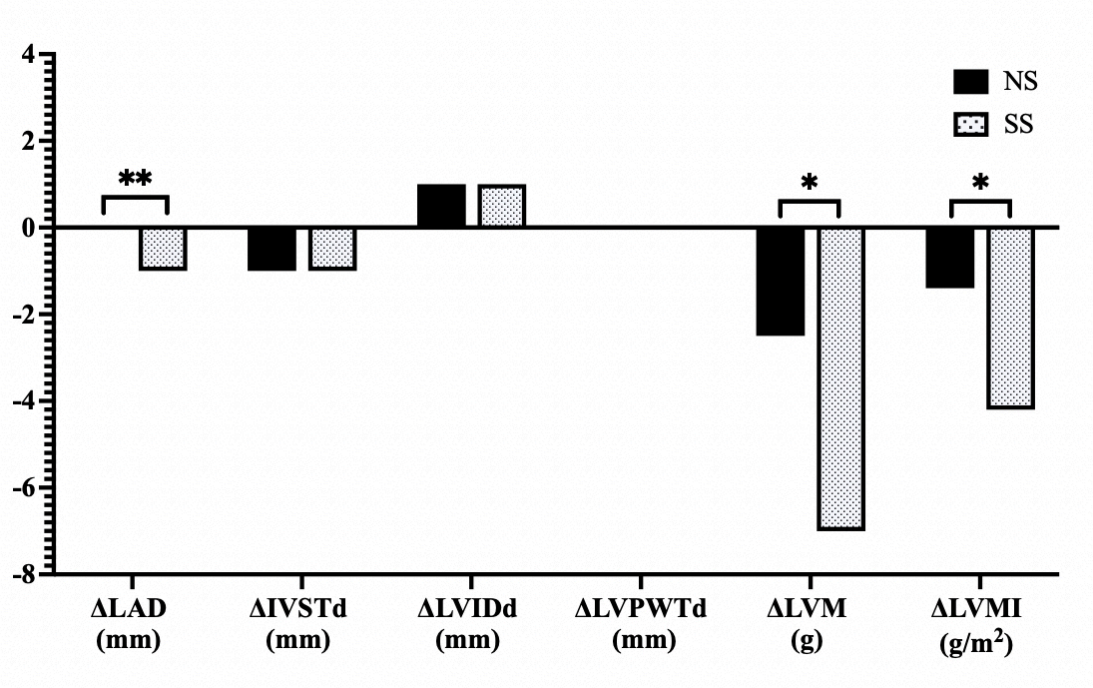
**Comparison of changes in cardiac structural parameters in response to SS diets in hypertensive patients**. NS, normal salt; SS, salt substitution; LAD, left atrial dimension; IVSTd, interventricular septal thickness at end diastole; LVIDd, left interventricular internal diameter at end diastole; LVPWTd, left interventricular posterior wall thickness at end diastole; LVM, left ventricular mass; LVMI, left ventricular mass index; Δ: net difference, * *p* < 0.05, ** *p* < 0.001.

Treatment with SS induced significant differences in the measurements of LAD (–1 mm [–2, 1] versus 0 mm [–1, 1], *p* < 0.001), LVM (–7.0 g [–18.0, 6.0] versus –2.5 g [–18.0, 11.0], *p* = 0.035), and LVMI (–4.2 g/m^2^ [–9.7, 3.4] versus –1.4 g/m^2^ [–10.1, 6.1], *p* = 0.041) when compared to the NS group. There were no significant differences between treatments in the measurements of IVSTd (–1 mm [–1, 0] versus –1 mm [–1, 0], *p* = 0.194), LVIDd (1 mm [–1, 1] versus 1 mm [–1, 1], *p* = 0.535), or LVPWTd (0 mm [–1, 1] versus 0 mm [–1, 0.75], *p* = 0.239). These findings suggest that the salt substitution may have a more pronounced effect on certain cardiac structural parameters (Fig. 4).

### 3.5 Electrocardiogram Parameters at Baseline and the Endpoint

**Fig. 5.**
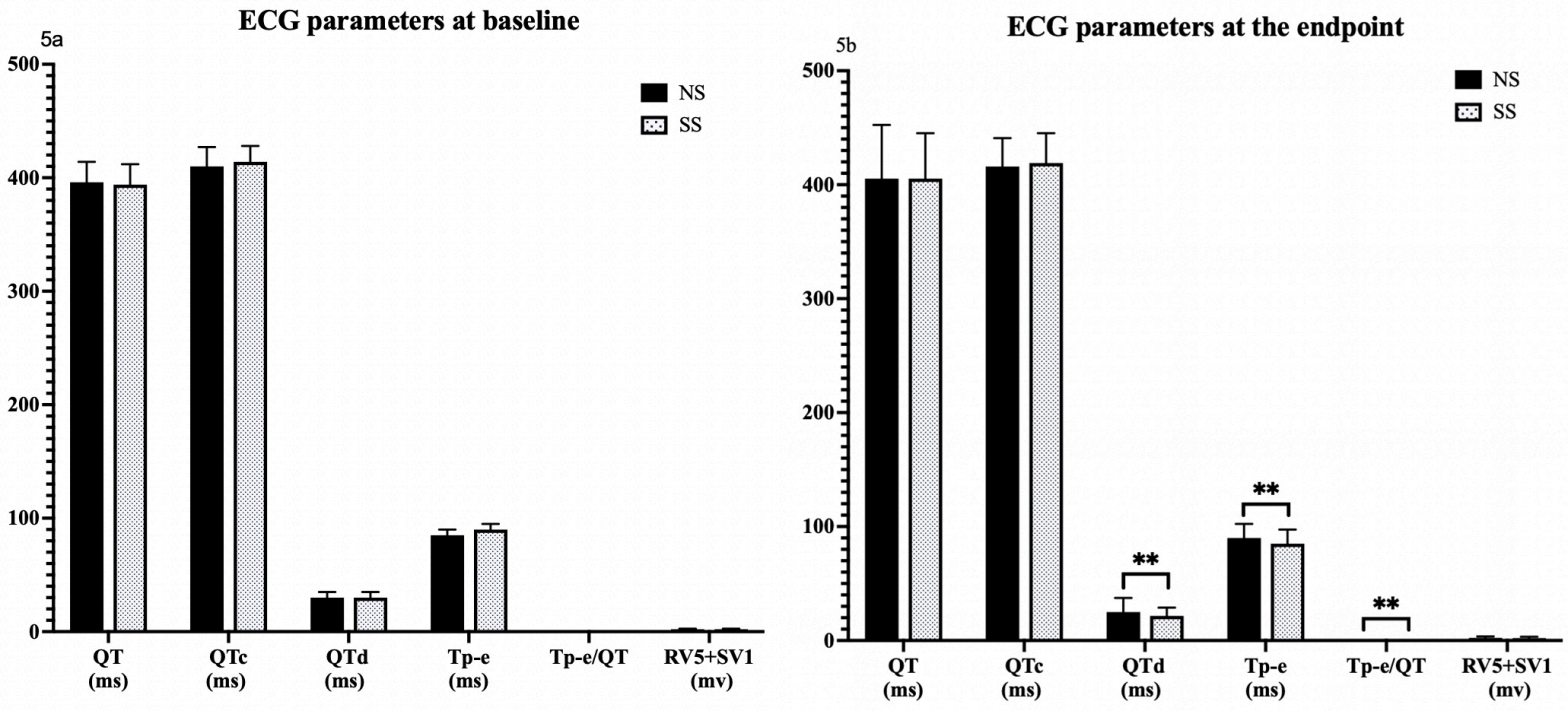
**Differential impact of salt substitution on ECG parameters at study baseline and endpoints**. Comparison of ECG parameters (QT, QTc, QTd, Tp-e, Tp-e/QT, RV5+SV1) between NS and SS groups at baseline (5a) and the endpoint (5b). ** *p* < 0.001. NS, normal salt; SS, salt substitution; ECG, electrocardiogram; QT, QT interval; QTc, corrected QT interval; QTd, QT dispersion; Tp-e, T wave peak-to end; RV5+SV1, sum of the amplitudes of the R wave in lead V5 and the S wave in lead V1.

At baseline, ECG parameters including QT, QTc, QTd, Tp-e, Tp-e/QT, and R wave voltage in lead V5 (RV5) + S wave voltage in lead V1 (SV1) showed no differences between the NS and SS groups. However, at the study endpoint, significant reductions were observed in the SS group compared to the NS group for QTd (20 ms [20, 25] versus 25 ms [20, 30], *p* < 0.001), Tp-e (85 ms [80, 90] versus 90 ms [85, 95], *p* < 0.001), and Tp-e/QT ([0.19, 0.22] versus 0.22 [0.21, 0.24], *p* < 0.001 for Tp-e/QT). No significant changes were found in QT, QTc, or RV5 + SV1 between the two groups (Fig. 5).

### 3.6 Comparison of Changes in ECG Parameters between the NS and SS Groups

**Fig. 6.**
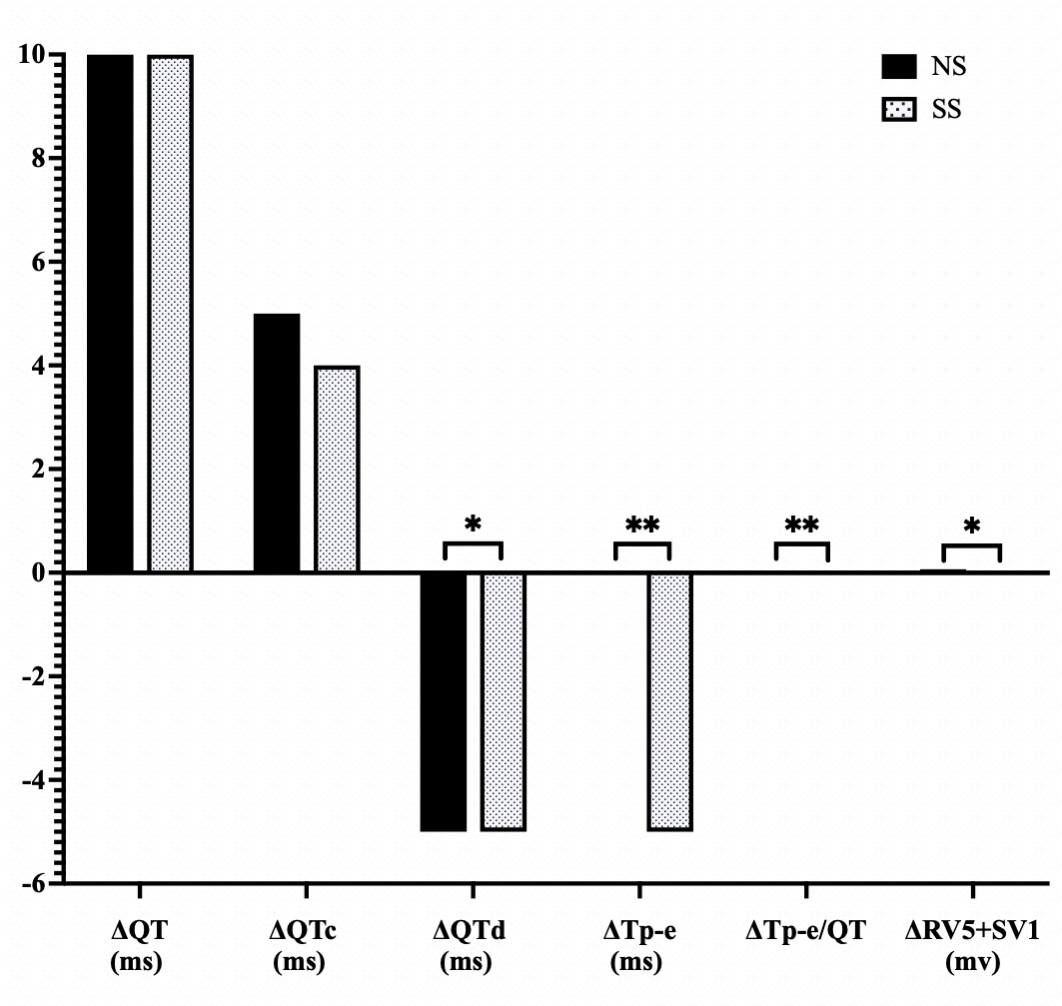
**Comparison of changes in ECG parameters (endpoint to baseline) in response to SS diets in hypertensive patients**. NS, normal salt; SS, salt substitution; ECG, electrocardiogram; QT, QT interval; QTc, corrected QT interval; QTd, QT dispersion; Tp-e, T wave peak-to end; RV5+SV1, sum of the amplitudes of the R wave in lead V5 and the S wave in lead V1; Δ: net difference, *p < 0.05, ** *p* < 0.001.

We next examined the changes in ECG parameters between endpoint and baseline. There were no significant differences in the changes of QT or QTc between the two groups (*p* = 0.73 and *p* = 0.486, respectively). However, the SS diet did induce changes to QTd ( –5 ms [–10, 5] vs –5 ms [–15, 0], *p* = 0.001), Tp-e (0 ms [–5, 10] vs –5 ms [–10, 0], *p* < 0.001), and Tp-e/QT (0 [–0.01, 0.02] vs –0.02 [–0.04, 0], *p* < 0.001), when compared to patients on a NS diet. Additionally, the change in RV5 + SV1 was 0.07 mv (–0.12, 0.33) in the NS group compared to 0 mv (–0.21, 0.2) in SS group (*p* = 0.014) (Fig. 6).

## 4. Discussion

This study demonstrated significant antihypertensive effects of a SS diet in both OBPM and HBPM for both SBP and DBP when compared to participants consuming a NS diet. Furthermore, SS significantly improved cardiac structure and UCG parameters in middle-aged and elderly hypertensive patients. Our current results align with previous studies indicating that SS lowers blood pressure and reduces cardiovascular events among elderly residents in care facilities [13]. These results, utilizing a larger sample size, represent an update of previous data from our lab where the OBPM change in DBP did not reach statistical significance [12]. These results underscore the robustness of SS as a therapeutic strategy.

While a positive relationship was found between sodium intake and LAD [14], historical data has previously yielded mixed results. In 1989, Cuneo *et al*. [15] observed no significant change in mean LAD after six days of elevated sodium intake (approximately 220 mmol/d), though a subsequent infusion of 2 L of saline within 2 h on the seventh day increased LAD. Conversely, Rush *et al*. [16] found a significant reduction in LAD in dogs on a low-sodium diet compared to those on a high-sodium diet. In our study, the first to investigate the effect of SS on LAD in hypertensive patients, a significant decrease in LAD was noted in the intervention group compared to the control group, highlighting the sensitivity of LAD as an early indicator of hypertension effects in response to dietary sodium adjustment.

Although no significant differences were found in other cardiac structural parameters such as IVSTd, LVIDd, and LVPWTd, the significant change in LAD was anticipated. Left atrial enlargement, as detected by UCG, is recognized as an early indication of hypertension [17], supporting the observed reduction in LAD following SS treatment. Ferrara *et al*. [18] similarly reported a significant reduction in LVM after just six weeks of rigorous salt restriction, where sodium intake was notably reduced to about 1016 mg per day. However, another clinical trial did not observe LVM reduction in patients after 12 months of lower dialysate sodium during hemodialysis [19]. These differing outcomes highlight the variability in response, which can be attributed to differences in study populations, intervention methods, degrees of salt restriction, and study durations.

To the best of our knowledge, this study is the first to evaluate the impact of SS on ECG parameters. A previous study including 64 participants demonstrated that a low-salt diet significantly decreased corrected QTd and Tp-e values in normotensives within seven days, whereas these indexes were prolonged after 7 days of a high-salt diet as compared to a low-salt diet [20]. These changes were reversed with potassium supplementation, suggesting that sodium restriction and potassium enrichment can beneficially modify ECG parameters [20]. However, the earlier study was limited by its small sample size and brief intervention period. In contrast, our findings indicate reductions in QTd, Tp-e, and Tp-e/QT ratios after 12 months of using a low-sodium high-potassium salt. Notably, Tp-e and Tp-e/QT ratios are critical markers for risk stratification in patients with Brugada syndrome [21] and have been proposed to predict malignant ventricular arrhythmia [22], underscoring the clinical importance of our results.

## 5. Strength and Limitations

Our study provides valuable insights into the effects of salt substitution on OBPM, HBPM, cardiac structure, and ECG parameters. As a result, it is reasonable to propose that SS be widely used. More clinical studies are needed to test the protective effect of SS on target organs, in addition to BP reduction. However, several limitations warrant consideration. While our study highlights the beneficial effects of salt substitution on OBPM, HBPM, cardiac structure, and ECG parameters, several limitations warrant further consideration. First, only middle-aged and elderly hypertensive patients were included in this study. A more diverse age range might provide a more comprehensive understanding of SS’s hypotensive and organ-protective benefits. Second, we enrolled only patients with hypertension who did not have severe renal dysfunction, so we did not examine whether salt substitution is feasible in patients with severe renal dysfunction. Third, the population was limited to northern China. Including patients from other geographic, cultural, and economic areas can validate the generalizability of these findings. Fourth, dietary compliance was not directly monitored. The degree of compliance can affect the reliability of the study’s conclusions about the efficacy of SS. Future studies should address these gaps to enhance the generalizability and applicability of SS in diverse patient populations.

## 6. Conclusions

In summary, this study demonstrated that SS had a significant impact on cardiac structure and ECG parameters, in addition to BP reduction. Despite these benefits, not all hypertensive patients demonstrate a connection between their salt intake and hypertension, and some may resist dietary changes [23]. Therefore, effective strategies are needed to clarify the connection between salt intake and hypertension and to promote salt reduction. These strategies could include emphasizing the benefits of reducing sodium intake or decreasing the consumption of processed and ultra-processed foods [24].

## Availability of Data and Materials

The data sets generated and analyzed during the current study are not publicly available but are available from the corresponding author on reasonable request.
